# Evaluation of a transbronchial cryoprobe for the ablation of pulmonary nodules: an in vitro pilot study

**DOI:** 10.1186/s12890-023-02358-y

**Published:** 2023-02-22

**Authors:** Dániel Hammer, Lilla Büdi, Ádám Nagy, Rita Varga, Péter Horváth

**Affiliations:** Department of Pulmonology, Semmelweis University, Tömő utca 25-29, H-1083 Budapest, Hungary

**Keywords:** Transbronchial, Cryoablation, Lung, Dual-freeze protocol, Triple-freeze protocol

## Abstract

**Background:**

Ablation of malignant pulmonary nodules is a novel therapeutic option for patients who cannot undergo surgery. Current transthoracic approaches cause pneumothorax and/or bleeding in a significant number of cases.

**Objective:**

Our purpose with this study was to evaluate cryoablation under in vitro conditions with a commercially available cryosurgery system.

**Methods:**

We used ballistic gelatin to model the thermal conduction of lung tissue. The cryoprobe was inserted in the ballistic gelatin with two thermal sensors, they were placed 0.5 cm and 1.0. cm from the probe, respectively, temperature was measured on both sides. We used single-, double- and triple-freeze protocols to see if we could freeze it to -20 °C.

**Results:**

We achieved − 18.6 ± 3.26 °C on the closer sensor (sensor 1) and − 3.7 ± 4.61 °C on the sensor further away (sensor 2) after 15 min using the single-freeze protocol. Using the dual-freeze protocol, we achieved − 23.2 ± 2,23 °C on sensor 1 and − 16.5 ± 2.82 °C on sensor 2. With the triple-freeze protocol we obtained − 23.5 ± 2.38 °C on sensor 1 and − 19.05 ± 3.22 °C on sensor 2.

**Conclusion:**

With dual-freeze, values above − 20 °C were achieved using nearer sensor data, but a plateau phase occurred as with continuous freezing. Using triple freeze, we reached − 20 °C at a distance of 0.5 cm from the probe, but not at 1 cm; therefore, we did not expand the diameter of the predicted necrosis zone.

**Supplementary Information:**

The online version contains supplementary material available at 10.1186/s12890-023-02358-y.

## Background

In the treatment of early-stage non-small cell lung cancer, we aim for radical surgery, as it is the only curative treatment modality [1]. The operability of the tumour depends on the clinical stage, the ECOG (Eastern Cooperative Oncology Group) performance state, age, comorbidities, the expected postoperative FEV_1_ (forced expiratory volume in first second), VO_2max_ (maximal oxygen consumption) and DL_CO_ (diffusing capacity for carbon monoxide) values of the patient and, last but not least, patient consent. It is not uncommon that surgical resection is technically feasible; however, due to comorbidities (e.g. COPD [chronic obstructive pulmonary disease] in smokers), it could not be tolerated because the VO_2max_ decrease after the lobectomy could be as high as 20% compared to the preoperative value [2].

As an alternative for surgical procedures, there are percutaneous ablation techniques (radiofrequency ablation [RFA], microwave ablation (MWA), and cryoablation), which are less invasive and involve less lung loss, so patients with impaired lung function may benefit from these [3]. Percutaneous cryoablation has its own potential complications, such as haemoptysis and pneumothorax [4]. In the last couple of years there were several attempts to perform ablation techniques with a transbronchial approach. This method would have the advantage of reducing the incidence of complications mentioned above, as this would not compromise the integrity of the thoracic wall and pleural surfaces [5, 6]. The results are promising, for example in this clinical study [7] 25 patients were treated with MWA via navigation bronchoscopy, who were ineligible for surgery. Technical success rate was 100%, the most common complication were pain (13.3%), and only two patients had pneumothorax. After median follow up of one year, none of the ablated nodules had evidence of progression.

Kohno et al. have formulated the properties that a future transbronchial cryoprobe should have: (1) a flexible probe tip that allows orotracheal penetration and can be easily guided into the target bronchus through the working channel of the bronchoscope, (2) a probe tip that allows bronchial passage into the tumour located in the parenchyma, (3) have sufficient freezing capacity to reach the minimum temperature of -20ºC that causes tissue necrosis, and (4) be leak-proof to avoid damage to healthy tissue [8].

In most studies, an argon-helium based probe is used with a rigid probe and a percutaneous approach [9, 10]. In a 2019 study [11], Surtees et al. compared the results of a single-component transthoracic carbon-dioxide probe, which is cheaper than argon-helium-based systems, with those of a classical argon-helium-based probe. Here again, the goal was to achieve a minimum of -20 °C, which was theoretically possible based on the Joule-Thompson effect. Their results and conclusions show that a carbon dioxide-based cryoablation system can achieve the anti-tumour effect of argon-based systems.

If we can achieve a freezing temperature of -20 °C around a flexible, transbronchial probe, it may even be a viable alternative to an argon-based cryoprobe system and thus suitable for the treatment of malignant lesions using a transbronchial approach, when surgery is contraindicated for early-stage cancers due to patient age, co-morbidities (often COPD) or other reasons.

The aim of our study was to investigate the freezing effect of a carbon dioxide-based flexible cryoprobe for tissue sampling and airway clearance at the Department of Pulmonology, Semmelweis University. We aimed to test different freezing protocols in a synthetic lung model.

## Materials and methods

### The synthetic lung model

Our aim was to create a simple but reliable lung model. In our measurements we used ballistic gelatin to model lung tissue, which had been previously reported to be suitable for this purpose, as the synthetic gelatin model is an alternative to animal lung models in terms of tactility, Hounsfield density and physical density [12]. Another study came to a similar conclusion by investigating thermal conductivity [13].

In the preparation of the ballistic gelatin, we followed the manufacturer’s instructions. (Dark Tactical, Budapest, Hungary) We divided the gelatin into Falcon tubes (50 mL, with a diameter of 29 mm and a length of 114 mm). All tubes with the ballistic gelatine were used only once.

### Thermometer

**Fig. 1:**
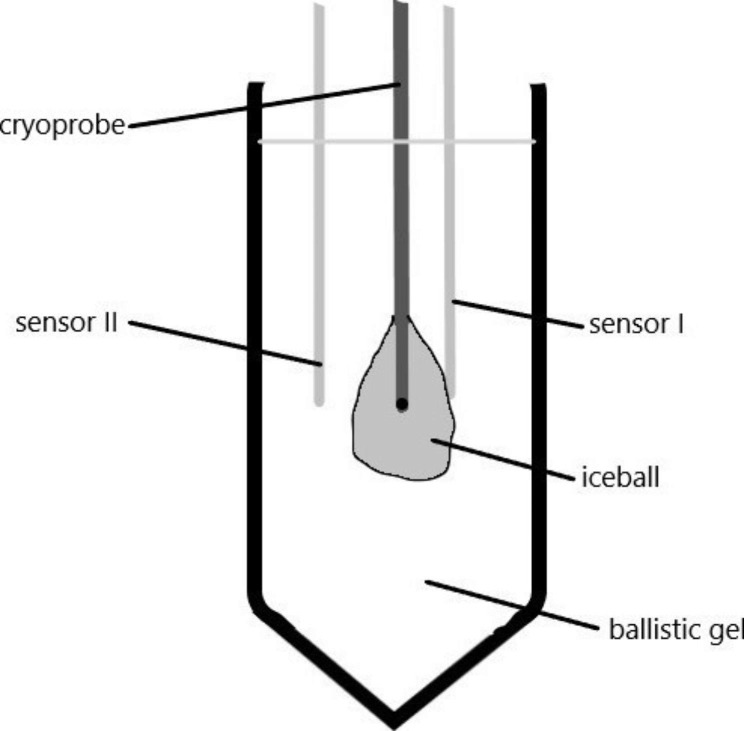
The set-up of our lung model, and the positions of the sensors and the cryoprobe

Thermometers of type DS18B20 (SenstechLtd, Shenzhen, China) were inserted into the ballistic gelatin, and the detected data were recorded by an Arduino Uno microprocessor [14]. Sensor 1 was placed 0.5 cm from the probe, sensor 2 1 cm from the probe. The setup can be seen on Fig. 1. Data were recorded using the R programming language on a laptop connected to the system. (For the source code, see supplementary material.) A photograph of the setup can be seen as supplementary material.

### Cryoprobe

For our measurements, we used the ERBECRYO® 2 (ErbeElektromedizin GmbH, Item No. 10402-000) system with a flexible 2.4 mm diameter probe (ErbeElektromedizin GmbH, Item No. 20402-032). This type of probe is recommended by the manufacturer to be used for a total of 15 min of freezing at a time. The cooling energy was obtained from a carbon dioxide cylinder.

### The single-, dual- and triple-freeze protocols

The manufacturer recommendation is to use their cryoprobe for a continuous maximum of 15 min, therefore we did our first experience with 15 min of continuous freezing.

For the dual-freeze protocol we used the same protocol as Hinshaw et al. in their study [15], because our own results with the continuous freezing suggested, that after 8 min of freezing, there is a plateau phase, where the temperature did not decrease significantly.

During the triple freeze procedure, after 8 min of freezing, we let the gelatin warm up for 3 min, followed by another 8 min of the freezing cycle, then another 3 min of warming up, and at the end we applied 6 min of freezing. We chose the length of the freezing cycles based on our previous results, as the plateau phase occurs after 8 min of freezing. Patrick J et al. previously used 3 min of passive thawing in their study as well [16].

### Statistical analysis

Results were analysed using the R programming language. (R Statistical Foundation, Vienna, Austria). All measurements were evaluated by ANOVA. Bonferroni correction was used for post hoc analysis. Where only two measurement points were compared, Welch’s t-test was used. The value of α was taken as 0.05. Results were plotted on graphs using the R language ggplot2 package [17].

## Results

### Fifteen minutes of continuous freezing

We tested whether the probe could reach − 20 °C during 15 min of continuous freezing.

Our measurements (n = 12) show that there is a significant difference between the two sensors after the first minute. The temperatures measured begin to approach the plateau phase after the 8th minute on sensor 1 and on sensor 2 after the 5th minute (significance is lost between the minute-by-minute temperature readings).

**Fig. 2:**
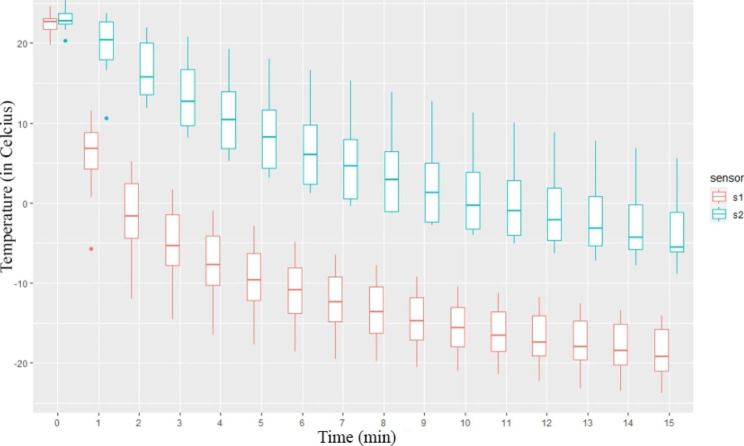
Graph of 15-minute continuous freezing in Celsius/min (n= 12)

Sensor readings closer to the cryoprobe indicate that the target necrosis-inducing temperature of -20 °C was reached several times but was only approached on several occasions. At the end of the measurement, we reached − 18.6 ± 3.26 °C with the closer sensor 1 and − 3.7 ± 4.61 °C with sensor 2, which was further away. A graphical representation of the measurement results is shown in Fig. 2.

### Testing the dual-freeze protocol

After 15 min of continuous freezing, we investigated whether the use of a dual-freeze protocol with passive thawing yielded significantly different results compared to continuous freezing (n = 7). As in the previous experiment, the sensors (1 and 2) were placed 0.5 and 1 cm from the cryoprobe tip in the gelatin, respectively.

The two sensors measured almost the same room temperature in the gelatin at the initial moment. As expected, sensor 1 measured lower values during the experiment than sensor 2, which was further away. At the end of the 25th minute, sensor 1 and sensor 2 measured − 23.2 ± 2.23 °C and − 15 ± 4.74 °C respectively. As in the previous experiment, the difference between the values obtained with the probe that was further away was larger.

**Fig. 3:**
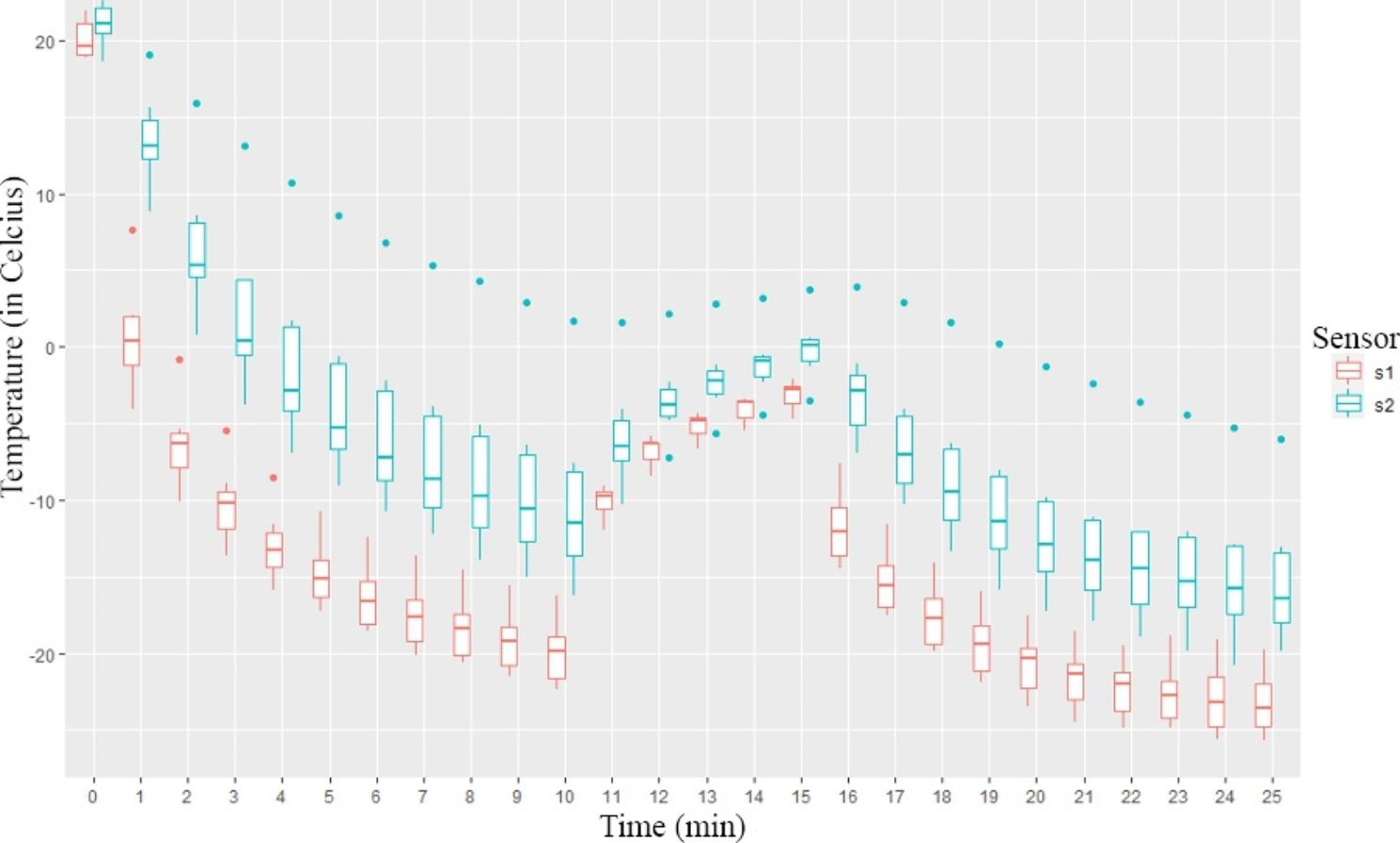
Graph of the results of the dual-freeze measurement in Celsius/min. The sensor marked s1 was positioned 0.5 cm from the cryoprobe, and s2 was positioned 1 cm from the cryoprobe. (n= 7)

When comparing the endpoints of the first and the second freezing cycles, we can see a significantly lower temperature at sensor 1 (Ts1 = -19.9 ± 2.18 °C vs. Ts1 = -23.2 ± 2.2 °C, at the 10th and 25th min, p = 0.02). On sensor 2, the difference in temperature is not significant (Ts2 = -9.9 ± 5.99 °C vs. Ts2 = -15.0 ± 4.7 °C, at minutes 10 and 25, p = 0.10). A graphical representation of the measurement results is shown in Fig. 3.

### Testing the triple-freeze protocol

**Fig. 4:**
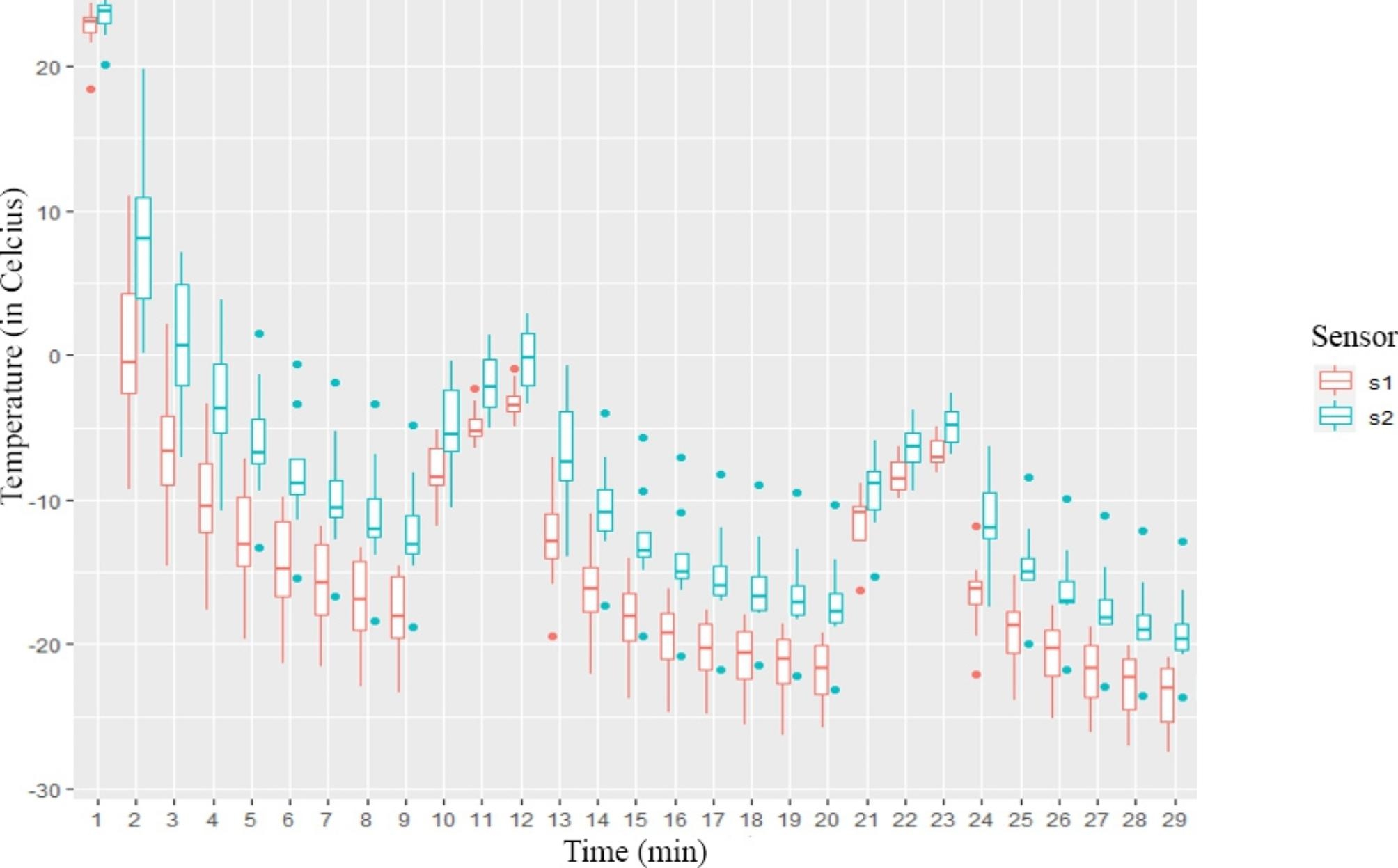
Graph of the results of the triple-freeze measurement in Celsius/min. The sensor marked s1 was positioned 0.5 cm from the cryoprobe, and s2 was positioned 1 cm from the cryoprobe. (n= 8)

After the dual-freeze method we tested the triple-freeze one (n = 8). Although we reached the − 20 °C at 0.5 cm from the cryoprobe in all measurements, we did not always reach it at 1 cm. Moreover, there was no significant difference between the data measured on the same sensor at the end of the second and third freezing periods. However, sensor 1 measured significantly lower values after all freezing cycles. (Ts1 = -22 ± 2.48 °C, Ts2 = -17.2 ± 3.72 °C at the end of the second freezing period (p = 0.01); Ts1 = -23.5 ± 2.38 °C, Ts2 = -19.05 ± 3.22 °C at the end of the third freezing period p = 0.01). A graphical representation of the measurement results is shown in Fig. 4.

## Discussion

According to the literature, cryoablation is an effective alternative for the treatment of functionally inoperable patients. Haemorrhage and inflammation following freezing, when used in combination with immunotherapy, can have a synergistic effect in the treatment of tumours [18]. With a flexible cryoprobe, we expect a significant reduction of potential side effects in clinical practice (pneumothorax, and pleural bleeding). Given the availability of flexible CO2-based cryoprobes, the practical uptake of this technique may be significantly faster than that of flexible thermocoagulation catheters.

We began our experiments by testing whether 15 min of continuous freezing in our lung model would achieve − 20 °C causing necrosis of lung tumours. We set the time to 15 min because the probe manufacturer recommended a maximum of 15 min in a row, and Hinshaw et al. used a total of 15 min of active freezing, albeit interspersed with thawing, in their triple-freeze protocol [15]. Surtees et al. used a rigid carbon dioxide probe to reach − 40 °C in the immediate vicinity of the probe, well below the desired target temperature, and their measurements suggest that the probe may be suitable for clinical use in practice [11].

On the basis of our measurements, the temperature did not always reach the minimum of -20 °C with continuous freezing, although we did exceed this threshold several times within 0.5 cm of the catheter.

As freezing in lung tissue should be interrupted by at least one thawing [19, 20], we also investigated the dual-freeze protocol in our experimental system.

At the end of the 25-minute measurement, the target value of -20 °C was reached and exceeded in all but one measurement. This can be explained by the fact that the gelatin around the probe was frozen, and the thermal conductivity of ice is significantly better than that of water or air.

This may explain the 18 °C temperature rise observed by sensor 1, but it returned to pre-melting values in the 5th minute of the new freeze cycle again.

During continuous freezing, values higher than − 20 °C were observed at the end of the procedures with sensor 2, causing tissue devitalization. These results suggest that the probe is not suitable for devitalization of tumour masses with radii larger than 0.5 cm using the single-freezing protocol. The dual-freeze protocol also approached temperatures close to -20 °C on sensor 2, suggesting that the devitalization zone achievable with the dual-freeze protocol was somewhere between 1 and 2 cm in diameter.

With the triple-freeze protocol we did not reach significantly lower temperature levels after the third cycle. The ablation zone could be increased by using a probe with a wider diameter, however there is a limitation here due to the size of the bronchoscopes’ working channel. The biggest therapeutic bronchoscopes have a 3.2 mm working channel, so the maximal diameter for the probe is approximately 3 mm. Based on the Joule-Thompson curve of different gases, we could choose another gas, like dinitrogen-oxide, nitrogen or argon. The cryosystem available to us was designed to be used with carbon-dioxide, therefore we could not test the freezing properties of other gases.

Our results with the flexible cryoprobe are in contrast to those previously reported by other authors using argon-helium-based systems. This may be due to the fact that the Joule-Thomson curve of carbon dioxide gas is shifted to the right compared to argon, requiring less energy to increase the volume per unit volume.

At the beginning of our measurements, the gelatin we used was at near room temperature, which is below the temperature expected in human living tissue of approximately 36 °C. In the present cycle, we did not investigate whether a higher starting temperature had an effect on the results obtained at the end of the measurements. However, on the basis of the poor thermal conductivity of air-filled lung tissue, it is conceivable that rewarming after the first freezing cycle would not be significantly more pronounced, nor would the presence of large calibre blood vessels running nearby be expected in peripheral lesions.

Our study has its limitations. First of all it is an in vitro study, therefore our results should be replicated in animal lung models. We began the freezing cycle from room temperature, therefore real life results might have higher temperature values. Also, we only tested carbon-dioxide, however there are other suitable gases available.

In conclusion, the system under investigation theoretically allows cryoablation of small T1A stage tumours. However, further experiments in animal models are needed to define the cryoablation zone precisely.

## Conclusion

This pilot study was conducted to investigate whether the carbon dioxide cryoprobe available at the Department of Pulmonology, Semmelweis University, could be suitable for the treatment of patients with inoperable lung cancer.

With dual-freeze, values above − 20 °C were achieved using nearer sensor data, but a plateau phase occurred similar to continuous freezing. Using triple-freeze, we reached − 20 °C at 0.5 cm from the probe, but not at 1 cm; therefore, we did not expand the diameter of the predicted necrosis zone.

These results suggest that, although our results approach the desired temperature, they do so only within a radius of 0.5 cm of the probe. At 1 cm, the dual- and triple-freeze protocols do not reach − 20 °C.

## Electronic supplementary material

Below is the link to the electronic supplementary material.

Supplementary Material 1

Supplementary Material 2

Supplementary Material 3

Supplementary Material 4

## Data Availability

Data are available as supplementary material.
