# Supplementary figures and images for: Evaluation of a transbronchial cryoprobe for the ablation of pulmonary nodules: an in vitro pilot study
Source: BMC Pulm Med. 2023 Feb 22;23:71. doi: 10.1186/s12890-023-02358-y (PMC9948372; doi:10.1186/s12890-023-02358-y)

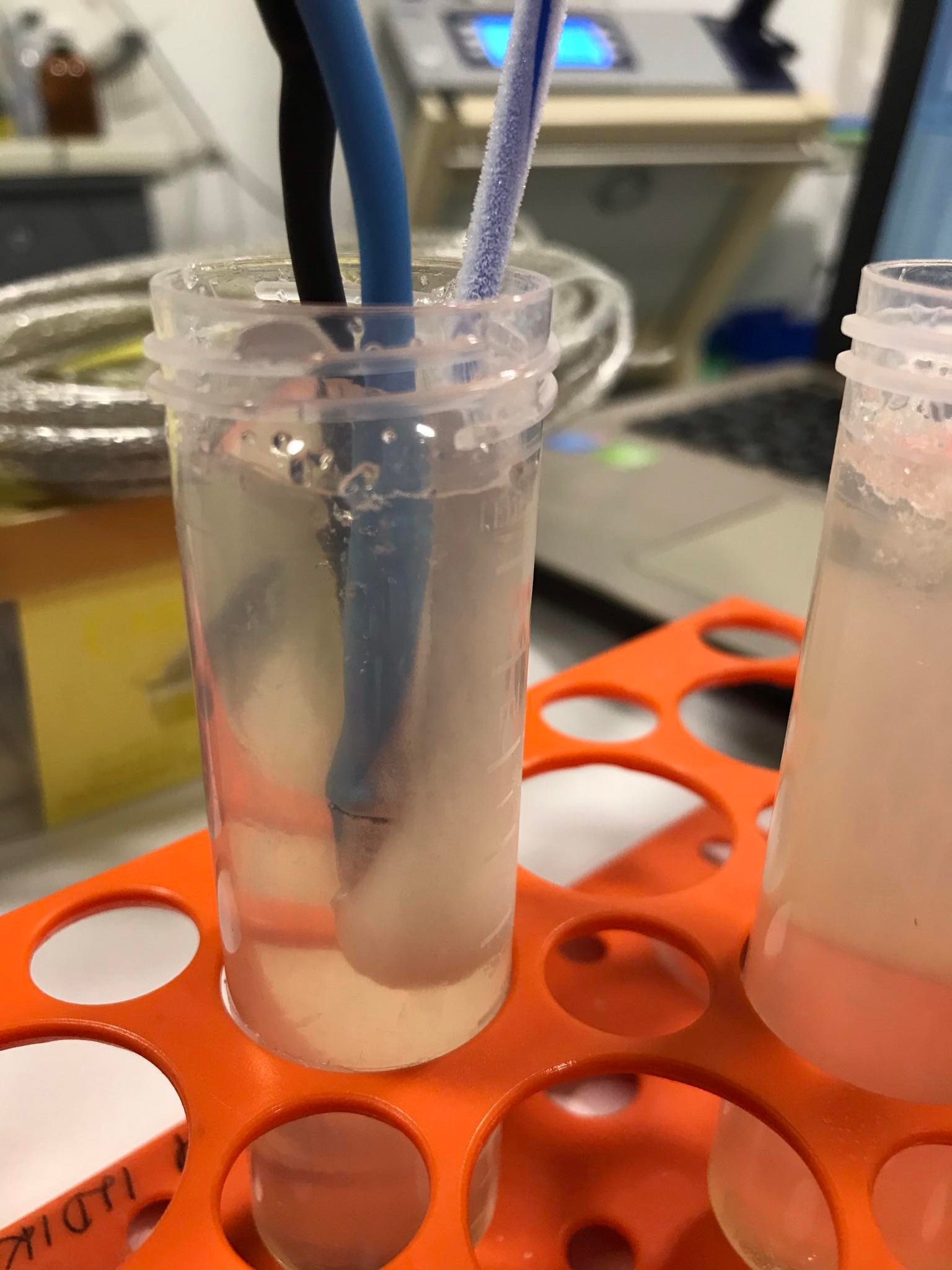

Supplement: Supplementary file 2 — Supplementary Material 2 [file 12890_2023_2358_MOESM2_ESM.jpg]
